# Trends in the Treatment of Hypertension from the Perspective of Traditional Chinese Medicine

**DOI:** 10.1155/2013/275279

**Published:** 2013-06-26

**Authors:** Xingjiang Xiong, Xiaochen Yang, Wei Liu, Fuyong Chu, Pengqian Wang, Jie Wang

**Affiliations:** ^1^Department of Cardiology, Guang'anmen Hospital, China Academy of Chinese Medical Sciences, Beixiange NO. 5, Xicheng District, Beijing 100053, China; ^2^Department of Cardiology, Traditional Chinese Medicine Hospital of Beijing, Beijing 100010, China; ^3^Department of Endocrinology, Traditional Chinese Medicine Hospital of Mentougou District, Beijing 102300, China

## Abstract

Hypertension is a major public-health issue. Much consensus has been reached in the treatment, and considerable progress has been made in the field of antihypertensive drugs. However, the standard-reaching rate of blood pressure is far from satisfaction. Considering these data and the seriousness of the effects of hypertension on the individual and society as a whole, both economically and socially, physicians must look for more effective and alternative ways to achieve the target blood pressure. Could treatment of hypertension be improved by insights from traditional Chinese medicine? As one of the most important parts in complementary and alternative therapies, TCM is regularly advocated for lowering elevated blood pressure. Due to the different understanding of the pathogenesis of hypertension between ancient and modern times, new understanding and treatment of hypertension need to be reexplored. Aiming to improve the efficacy of Chinese herbal medicine in treating hypertension, the basis of treatment is explored through systematically analyzing the literature available in both English and Chinese search engines. This paper systematically reviews the trends in emerging therapeutic strategies for hypertension from the perspective of traditional Chinese medicine.

## 1. Introduction

Hypertension is an increasingly important medical and public-health issue [1]. The prevention and management of hypertension are major public-health challenges. Many cardiovascular diseases (CVDs), cerebrovascular diseases, and hypertension would be preventable if the rise in blood pressure (BP) with age can be prevented or diminished [2]. Complementary and alternative medicine (CAM) therapies are increasingly popular and frequently used by patients with CVDs [3, 4]. Many CAM therapeutic options exist, and >95 CAM therapies have been recommended for hypertension [5]. Recent researches have shown that numerous CAM therapies are advocated for lowering elevated BP [5, 6]. Traditional Chinese medicine (TCM), including herbal medicine and acupuncture, is an important component of CAM therapies [7]. 

TCM played an important role in the provision of healthcare. Although there is no diagnosis of hypertension at ancient time in China, physicians attempted to treat hypertension-related symptoms by using TCM principles in clinical practice for centuries [8]. It is noteworthy that the poor antihypertensive efficacy of TCM has always been a major emerging problem, which has led to a high degree of consensus that Chinese medicines cannot independently treat hypertension [9–13]. However, considerable progress has been made in lowering BP by TCM recently [14–16]. Could treatment of hypertension be improved by insights from traditional Chinese medicine? Aiming to improve the efficacy of Chinese herbal medicine in treating hypertension, we explored the basis of treatment by systematically analyzing the literature available through both English and Chinese search engines, including the Cochrane Central Register of Controlled Trials (CENTRAL) in the Cochrane Library (September, 2012), MEDLINE (1959–2012), PUBMED (1959–2012), EMBASE, Chinese National Knowledge Infrastructure (CNKI) (1980–2012), Chinese Scientific Journal Database (VIP) (1989–2012), Chinese Biomedical Literature Database (CBM) (1978–2012), and WANFANG (1998–2012) databases, that discuss the potential uses of Chinese medicine therapies to treat hypertension. It was found that the aspects described below should be considered for the effective treatment of hypertension, and it is possible for TCM to be mainstream in health care system. The paper systematically reviews trends in emerging therapeutic strategies for hypertension from the perspective of traditional Chinese medicine. 

## 2. Disease-Syndrome Combination Is a New Mode for Treatment of Hypertension

Syndrome (also known as “pattern” or “zheng”) is the basic unit and a key concept in TCM theory, which has been used in China for over 2,500 years [17]. It is different from disease or symptom. TCM syndrome is the abstraction of a major disharmonious pathogenesis, which is identified from a comprehensive analysis of all symptoms and signs (tongue appearance and pulse feeling included) from four main diagnostic TCM methods: observation, listening, questioning, and pulse analyses [18, 19]. It is the generalization of cause, location, nature, and trend of disease in certain stage. In brief, all diagnostic and therapeutic methods in TCM are based on the differentiation of TCM syndrome [20, 21]. In ancient China, due to technological backwardness and lack of testing methods, Chinese practitioners explored the etiology and pathogenesis of a disease by conjecturing on the interior by observing the exterior; exploring the intrinsic etiology from extrinsic appearances; testing the state of the internal organs; and differentiating symptoms and signs. In general, it is believed that syndrome differentiation is a special feature and priority of TCM. Thus, syndrome differentiation plays an important role in the therapeutic process and affects the therapeutic result of certain diseases [22–24]. 

However, as mentioned above, syndrome is the outcome of differentiation of symptoms and signs, and definitely syndrome is relatively generous, vague, uncertain, and abstractive, which has brought great difficulties to the clinical and scientific research of TCM [17]. The following example can be used to explain the shortage of syndrome information. Hypertension, angioneurotic headache, and intracranial hypertension all could show similar symptoms and primary signs such as headache and dizziness, suggesting that they could be diagnosed as the same syndrome in TCM and could be treated by the same TCM approach. The effect, there is no doubt, should be different since the severity and prognosis of the above diseases are different. Thus, the differentiation of syndrome would not give any good effect when disease is not clarified based on biomedical diagnosis. More attentions have been paid to the treatment and the objective indicators of diseases. For example, BP, and blood levels of glucose and lipids as well as other objective indicators have become the focus of concern and assessment of therapeutic effects. Therefore, disease-syndrome combination, also called combination of syndrome classification and biomedical diagnosis, is a new mode for syndrome research involving the idea and theory of disease differentiation in western medicine as well as syndrome differentiation in TCM [25]. Currently, it has become a common model in the diagnosis and treatment of TCM clinical practice [26]. The mode of disease-syndrome combination has been successfully and widely applied to a variety of clinical diseases including restenosis after percutaneous coronary intervention [27], unstable angina [28], cancer [29], nonerosive reflux disease [30], rheumatoid arthritis [31], posthepatitis B liver cirrhosis [32], liver fibrosis [33], poststroke dysphagia [34], menopausal symptoms [35], and psoriasis vulgaris [36]. 

The new mode has changed the treatment philosophy of hypertension and thus improved the antihypertensive efficacy of TCM. It included two cases. The first one is diagnosis based on disease-syndrome combination. As compared with TCM syndrome criteria or BP criteria alone, hypertension could be diagnosed more accurately by using the combination of TCM syndrome and BP. When referring to Chinese medicine treatment alone based on diagnosis of disease-syndrome combination, Zhong et al. [37] conducted a randomized controlled trial to observe the effect of Chinese herbal medicine for calming *Gan* and suppressing hyperactive *yang* (CGSHY) on arterial elasticity function and the circadian rhythm of blood pressure in patients with essential hypertension (EH). The authors studied 64 patients comparing Chinese herbal medicine with enalapril to a control. After 12 weeks of treatment, significant difference was found in the ratio of T/P of SBP & DBP and in the levels of NO and ET-1 between treatment and control groups after treatment. The trial showed that Chinese herbal medicine for CGSHY may lower the blood pressure smoothly, recover the circadian rhythm of BP in EH patients, and improve the carotid elasticity of EH patients, which is similar to that of enalapril. 

The second one is treatment based on disease-syndrome combination. Previous studies have shown that TCM can improve syndromes and symptoms such as headache, vertigo, and fatigue (in particular for patients with liver *yang* hyperactivity syndrome or liver-kidney *yin* deficiency syndrome), whereas it cannot effectively improve objective indicators and pathological mechanisms such as BP, blood pressure variability (BPV), target organ damage, lipid disorders, insulin resistance, and leptin resistance [38–40]. Thus, it was concluded that Chinese herbs and formulas had poor efficacy for improving the objective indicators and pathological mechanisms of hypertension, leading to Chinese herbs and formulas being considered as “adjunctive treatment methods” [8]. Therefore, achieving the optimal target BP is a crucial issue in hypertensive patients currently. Numerous studies have confirmed that Chinese herbs and formulas could contribute to lowering BP smoothly [16]. While referring to combination therapy of Chinese medicine and western medicine based on treatment of disease-syndrome combination, another multicenter, randomized, double-blinded controlled trial was conducted by Li et al. [41]. A total of 270 cases were included to observe the effects of Chinese medical regimen and integrative medical regimen on quality of life and early renal impairment in elderly patients with isolated systolic hypertension (EISH). 3 groups were divided as Chinese medicine group (CM), combination group, and western medicine group (WM). After 12 weeks of treatment, the combination group was superior to CM or WM group in depressing systolic blood pressure, improving integral of quality of life, and decreasing the levels of mALB and *β*2-MG in urine. It was concluded that combination therapy has affirmative effect in treating EISH patient, and deserves further study. 

The disease-syndrome combination is not only the cornerstone of hypertensive treatment in TCM, but also an important way of TCM “merging” into modern clinical treatment. It is noteworthy that one must pay considerable attention to syndromes as well as the objective indicators for the treatment of TCM syndrome and disease together. However, clinical evidence of the efficacy of TCM on the mortality and morbidity of hypertension still needs to be strengthened in future researches.

## 3. A Different Understanding between “Vertigo and Headache” and Hypertension Is the Ideological Basis for the Treatment of Hypertension

According to TCM theory, hypertension belongs to the category of “vertigo and headache,” which has been recorded in the ancient Chinese medical literature such as *The Canon of Internal Medicine* and *Synopsis of Golden Chamber*. Therefore, TCM principles, which have been used to treat “vertigo and headache” in clinical practice for centuries, have been applied to the treatment of hypertension by physicians in China. However, it cannot be neglected that some new problems have arisen in treating hypertension due to the different understanding between “vertigo and headache” and hypertension. Previous treatment experience cannot solve these problems perfectly. Both specific differences between them and new understanding about the etiology, pathogenesis, diagnosis, and treatment of hypertension are needed to be explored.

Firstly, “vertigo and headache” is not the same as hypertension. It is due to the following four reasons: (a) diagnostic criteria: as the concept of BP has not yet appeared in ancient China, the diagnosis, clinical evaluation, and treatment of “vertigo and headache” were all based on “signs and subjective symptoms instead of BP” according to the unique concept of “wholism” in the understanding and treatment of the disease [42, 43]. While in modern medicine, BP and blood pressure variability (BPV) are regarded as the diagnostic gold standard of hypertension [44]. Although we cannot rule out the possibility that most of the patients with “vertigo and headache” have hypertension, but the diagnostic basis in TCM is incompletely understood. Patients who are diagnosed with vertigo and headache in TCM may possibly not meet the diagnosis of hypertension actually. (b) Diagnosis time: “Vertigo and headache” could be only diagnosed when uncomfortable symptoms appear. It cannot be excluded that some hypertensive patients are asymptomatic. However, as the measurement of BP was so simple and easy to operate, hypertension is diagnosed much earlier, even in asymptomatic period or prehypertension stage. (c) Usage of antihypertensive drugs: there is no intervention of conventional medicine for “vertigo and headache” in ancient time. However, hypertension is generally treated by antihypertensive drugs. (d) Control of BP: during the treatment of “vertigo and headache” in ancient time, the control of BP was still unclear. However, it could be well controlled by antihypertensive drugs in hypertension treatment. Therefore, we cannot structurally be consistent with the previous experience of “vertigo and headache” for hypertension [45]. 

Secondly, the pathogenesis of “vertigo and headache” has been changed and different pathogenesis between “vertigo and headache” and hypertension should also be paid attention to. In the ancient TCM literature, *Basic Questions of the Yellow Emperor's Inner Classic: Truth and Importance by the Theory,* the author states “the wind syndrome, such as convulsion, vertigo, and dizziness, all belong to the liver,” which showed that vertigo is closely related to the “liver and wind” according to TCM theory. Therefore, it has become the precursor of treating hypertension from the perspective of liver-yang and liver-wind [46]. Recently, studies also indicated that liver yang hyperactivity syndrome is a crucial syndrome of hypertension [47, 48]. Consequently, it is widely accepted that hypertension should be treated based on the theory of liver-wind until now [49]. Famous prescriptions for calming liver-wind including *Zhen gan xi feng decoction* (decoction for tranquilizing liver-wind) in *Yi Xue Zhong Zhong Can Xi Lu* (*Records of Chinese Medicine with Reference to Western Medicine*), *Tianma Gouteng Yin* (“decoction of *Gastrodia* and *Uncaria”*) in *Za Bing Zheng Zhi Xin Yi* (*New Meanings in Syndrome and Therapy of Miscellaneous Diseases*), and *Lingjiao gouteng decoction* (decoction of *Antelope Horn* and *Uncaria*) in *Chong Ding Tong Su Shang Han Lun* (revised popular guide to *Treatise on Febrile Diseases*) are all widely used today. However, some new issues have emerged due to great changes have been taken place for the treatment of vertigo. And considerable attention should be paid on the different pathogenesis between “vertigo and headache” and hypertension. These differences are discussed below. (a) The natural progression of “vertigo and headache” has been changed due to the following three reasons in modern medicine: early diagnosis, early intervention, and the continual optimization of medical methods. In particular, the old transformational principle of pathogenesis about “vertigo and headache,” that is, “the changes from excess of liver fire to liver yang hyperactivity, then to liver-kidney yin deficiency, finally to deficiency of both yin and yang,” has been interrupted [50, 51]. (b) Therapeutic function of antihypertensive drugs has potential influence on the pathogenesis of “vertigo and headache.” For example, beta blockers can be used to decrease heart rate; however, this action could also purge liver fire and heart fire to significantly improve fire syndrome in TCM. Therefore, it can lead to deficiency syndrome more easily due to the negative chronotropic action and negative inotropic effects [52]. It is worth noting that the number of patients with liver yang hyperactivity syndrome, hyperactivity of heart fire, and liver fire syndrome is decreasing, whereas the number of patients with deficiency syndrome (such as *qi* deficiency syndrome and kidney deficiency syndrome) is increasing [16, 51]. (c) The adverse effects of antihypertensive drugs can affect the pathogenesis of “vertigo and headache” directly. For instance, calcium-channel blockers have adverse effects such as hypotension, headache, facial flushing, polyuria, constipation, tibia, and ankle edema; *β*-blockers and diuretic could affect sexual function [53–56]. What is more, it was found out that patients who have used oral antihypertensive drugs for several years are more prone to suffer from waist soreness, tiredness in the loins and legs, and erectile dysfunction, which belongs to the category of fluid retention syndrome or kidney deficiency syndrome in TCM [57]. (d) Owing to changes of the disease spectrum, hypertension is associated with more comorbidities, thus leading to multisystem and multiorgan damage. With the increasing incidence of metabolic diseases, hypertension combined with diabetes mellitus and lipid abnormalities becomes quite common now [58, 59]. 

In summary, a different understanding between “vertigo and headache” and hypertension is the ideological basis for the treatment of hypertension. Due to the differences between “vertigo and headache” and hypertension, the pathogenesis law of hypertension is still unknown and should be reevaluated. 

## 4. Application of Herbal Pharmacological Achievements in Clinical Treatment Is an Important Reference for Lowering BP by Chinese Herbs and Formulas

In recent years, researchers have carried out several herbal pharmacology studies [60–62]. With continuous enrichment of achievements in herbal pharmacology, the effective components and pharmacological effects of many Chinese herbs and formulas have been illustrated [63], which provides evidence for the treatment of modern diseases [64]. Chinese herbs such as *Tianma* (Gastrodia) [65], *Gouteng* (Uncaria) [66], *Shijueming* (Abalone Shell), *Zhenzhumu* (Nacre or mother of pearl), *Daizheshi* (Ruddle), *Cishi* (Magnetite), *Juhua* (Chrysanthemum) [67], *Juemingzi* (Cassia seed), *Xixiancao* (Herba Siegesbeckiae) [68], *Baijili* (*Tribulus Terrestris* L.) [69], *Xiakucao* (*Prunella vulgaris* L.), *Luobuma* (*Apocynum venetum* L.), *Duzhong* (*Eucommia ulmoides*) [70–72], *Niuxi* (*Achyranthes* root), *Sangjisheng* (*Loranthus*) [73, 74], *Huangqi* (*Astragalus membranaceus*) [75–77], *Shengdihuang* (*Radix rehmanniae*), *Chuanxiong* (*Ligusticum Chuanxiong* Hort) [78], *Gegen* (*Kudzu* root) [79, 80], *Chishao* (red peony root), *Danshen* (*Salvia miltiorrhiza*) [81], *Yimucao *(*Leonurus japonicus*), *Shenghuaihua* (*Sophora* flower), *Chongweizi *(*Leonurus artemisia* (lour.) S.Y. Hu seed), *Shanzha* (Hawthorn) [82, 83], *Laifuzi* (radish seed) [67], *Huanglian* (*Coptis chinensis*), *Huangqin* (*Scutellaria baicalensis* Georgi), *Huangbai *(*Phellodendron* bark), *Zhizi* (Gardenia), *Xuanshen* (*Radix scrophulariae*), and *Lianzixin* (lotus Germ) can lower BP. In addition, other Chinese herbs such as *Dahuang* (*Rheum officinale* Baill.), *Heshouwu* (*Polygonum multiflorum* Thunb.), *Nvzhenzi* (*Ligustrum lucidum* Ait.), *Jinyingzi* (Cherokee rose), *Zexie* (*Alisma orientale*), *Juemingzi* (Cassia seed), and *Shanzha* (Hawthorn) lower levels of blood lipids to prevent atherosclerosis and coronary heart disease. 

Currently, two attitudes emerged about the application of herbal pharmacological achievements. The first one is rejecting. It is partially believed that TCM should lay special stress on the importance of “tradition” instead of “modern” in China. They reject all modern scientific achievements, including achievements of modern medicine. Therefore, due to the traditional habit of thinking, most achievements in herbal pharmacology have not been widely used in clinic treatment. Treatment remains reliant mainly on traditional clinical experience under the guidance of syndrome differentiation by TCM physicians. The second one is accepting. It is advocated by modern TCM practitioners that modern scientific achievements, such as modern medicine, biology, chemistry, physics, and genetics, should be made full use of in the process of modernization of TCM [50]. We are in favor of the latter view. Why Chinese herbs and formulas have poor antihypertensive efficacy? An important reason is that achievements in herbal pharmacology cannot be effectively converted to appropriate clinical use. It is thought that syndrome differentiation, the traditional therapy, has perfect effects in improving the signs, symptoms, and syndrome in TCM, but poor effects in treating disease (and even no effects in improving the key indicators of diseases) [84]. Therefore, application of the achievements of herbal pharmacology in clinical treatment is an important reference for lowering BP using Chinese herbs and formulas. 

However, some new problems have risen in the application of these achievements. Although some physicians have already consciously taken advantage of achievements in clinical treatment, most prescriptions are simple additions of these achievements, which violate the theory of syndrome differentiation in TCM. Therefore, how to appropriately apply herbal pharmacology achievements in the clinic without violating TCM theory should be considered carefully [50]. Our strategy is to combine syndrome differentiation and the achievements of herbal pharmacology together in clinical treatment. Clinical efficacy could be greatly improved if suitable herbs and formulas with clear therapeutic effects against disease were applied on the basis of the syndrome differentiation theory in TCM. It is noteworthy that some crucial Chinese herbs in these classical formulas (recommended for hypertension treatment in Table 1) definitely possess certain antihypertensive effect. All of these will be discussed as below.

According to our previous studies, hypertension could be divided into the following three major types on the basis of the stage and symptoms of the disease [8, 16]. Firstly, fire syndrome (e.g., liver fire, heart fire, stomach fire, and intestinal fire) can be found in various stages of hypertension, especially if target-organ damage is not found [16, 51]. When aiming to counteract liver fire syndrome, *Tianma Gouteng Yin* (decoction of *Gastrodia* and *Uncaria*), a famous prescription noted in *Za Bing Zheng Zhi Xin Yi* (*New Meanings in Syndrome and Therapy of Miscellaneous Diseases*), was recommended. It could suppress liver yang hyperactivity, clear heat, activate blood, and nourish the kidney. One systematic review (SR) also revealed that *Tianma Gouteng Yin* could contribute to lowering BP smoothly [85]. Moreover, Chinese herbs such as *Tianma* (*Gastrodia*), *Gouteng* (*Uncaria*), *Duzhong* (*Eucommia ulmoides*), and *Niuxi* (*Achyranthes* root) in the formula had good antihypertensive effects in pharmacological studies. Modern studies also showed that *Tianma Gouteng Yin* could improve memory, regulate the secretions of vasoactive substances, increase the serum concentration of nitric oxide (NO) and nitric oxide synthase (NOS), decrease levels of endothelin and angiotensin II, reduce the left ventricular mass index and collagen content, and improve left ventricular remodeling [86–88]. When aiming to counteract heart fire syndrome, *Huanglian Jie Du Tang* (detoxicant decoction of *Coptis*) was recommended [8]. It could clear heat and toxic materials and relieve headache and dizziness quickly. In addition, all the component drugs such as *Huanglian* (*Coptis chinensis*), *Huangqin* (*Scutellaria baicalensis Georgi*), *Huangbai* (*Phellodendron* bark), and *Zhizi* (*Gardenia*) could lower BP. Current researches also showed that *Huanglian Jie Du Tang* could control BP and improve the prothrombotic state in spontaneously hypertensive rats (SHRs) by decreasing the content of thromboxane (TX) A2, endothelin (ET)-1, homocysteine, and von Willebrand factor (vWF) and increasing the contents of 6-keto-prostaglandin and NO [89]. When aiming to counteract stomach fire and intestinal fire syndrome, *Zeng Ye Tang* (fluid-increasing decoction) was recommended [8]. It could not only nourish yin to relieve the symptoms of dryness but also lower BP by *Xuanshen* (*Radix scrophulariae*) and *Shengdihuang* (*Radix rehmanniae*) in the formula. 

Secondly, phlegm-fluid retention syndrome is another important type of hypertension in TCM, which is characterized by dizziness aggravated by change in body position; thirst without a desire to drink, or not being thirsty; chest distress; palpitation; gastric distension; abdominal distension; nausea; vomiting; poor appetite; lumbar heaviness; low back pain; weakness and heaviness in the lower extremities; edema; daytime sleepiness; abnormal leucorrhea; dysuria; and greasy fur [51]. It could be also divided into 2 syndromes: fluid retention syndrome and upward going of phlegm turbidity syndrome (UPTS). The former one could be treated by *Wuling* powder, *Zexie Tang* (decoction of *alisma*), and so forth. *Wuling* powder, a famous classical prescription recorded in *Shang Han Lun* (*Treatise on Febrile Diseases*) by *Zhang Zhongjing* in the Han Dynasty, was recommended [8]. It could not only remove dampness by promoting dieresis but also had satisfying therapeutic effects in increasing the discharge of urine, decreasing BP, and maintaining the balance of serum electrolyte contents in rats with renal hypertension [90]. *Zexie Tang* is another famous classical prescription used for treating fluid retention syndrome. It is effective in improving the construction and function of kidney injuries of rats with hypertension induced by a high-salt diet. This effect may be associated with increasing renin activity and angiotensin-II level in renal tissues, which thereby prevent kidney injuries [91]. *Zexie *(*Alisma*) and *Baizhu* (*Atractylodes*), the two component drugs in *Zexie Tang* (decoction of *alisma*), can lower the blood levels of lipids and glucose, respectively. While referring to the later one, fluid retention syndrome and liver-yang hyperactivity syndrome often appear simultaneously, which is known as UPTS. *Banxia Baizhu Tianma Tang* (decoction of *Pinellia ternata*, *Atractylodes macrocephala*, and *Gastrodia elata*) was recommended [8]. It could calm the liver, strengthen the spleen, and dissipate excessive fluid. One SR demonstrated that *Banxia Baizhu Tianma Tang* could contribute to lowering BP [92]. Among them, *Tianma* (*Gastrodia*), the monarch drug of the formula, is an important Chinese herb for lowering BP. Modern studies also showed that it can improve clinical symptoms, promote a stable decrease in BP, improve insulin resistance and salt sensitivity, decrease serum levels of total cholesterol (TC), triglycerides (TGs), and low-density lipoprotein-cholesterol (LDL-C), and regulate the rennin-angiotensin system (RAS) [93–95]. 

Deficiency syndrome is very common in hypertension [16, 51]. It is found that the longer the duration of illness, the higher the incidence rate of deficiency syndrome. When aiming to counteract kidney yin deficiency syndrome, *Liu Wei Dihuang Wan* (pill of *Rehmannia*) was recommended [8]. It could replenish kidney yin. One SR showed that *Liu Wei Dihuang Wan* could also contribute to hypertension treatment [96]. Among them, *Dihuang* (*Radix rehmanniae*) and *Danpi* (*Cortex moutan*) could lower BP. Studies have also shown that it can lessen damage to the heart and kidney in hypertensive patients by reducing the myocardial oxygen consumption index, reversing myocardial hypertrophy, improving left ventricular function, protecting the endothelium of blood vessels, preventing arteriosclerosis, increasing the glomerular filtration rate, and decreasing microalbuminuria [97, 98]. When aiming to counteract kidney yang deficiency syndrome, *Shen qi Wan* (kidney *qi* pill) was recommended [8]. It could not only recuperate the kidney yang but also improve the sexual function of hypertensive patients, reduce urinary levels of albumin, and protect renal function [99, 100]. 

Although application of herbal-pharmacology achievements to clinical treatment could contribute to disease-targeted treatment in TCM, the following aspects must be addressed. Firstly, as “formulas corresponding to syndromes” is a basic principle of TCM treatment, it is advocated that Chinese herbs and formulas which can not only treat the disease in western medicine but also TCM syndromes could be preferentially selected [50]. Hence, we cannot select Chinese herbs and formulas solely on the basis of their pharmacological effects in clinical treatment. For instance, there are many Chinese herbs for treating kidney deficiency syndrome; however, only *Duzhong* (*Eucommia ulmoides*), *Niuxi* (*Achyranthes* root), and *Sangjisheng* (Loranthus) should be given prior consideration for having a clear effect of treating hypertension and kidney deficiency syndrome together. Secondly, studies on the bioactive constituents of Chinese herbs cannot represent all the characteristics of Chinese herbs (especially the rules for clinical application). 

## 5. Reasonable Drug Dosage Is the Effective Way to Improve the Antihypertensive Efficacy of Chinese Herbs and Formulas

There exists an interesting phenomenon in clinical practice. That is, although some TCM physicians choose certain Chinese herbs and formulas with clear antihypertensive effects, antihypertensive efficacy remains difficult to improve. It may be related to the limitation of drug dose.

There are many factors that affect the clinical efficacy of TCM. These include an accurate diagnosis, accurate prescriptions, quality and dose of Chinese herbs, water decoction, and administration method. Among them, dosage remains the key issue restricting the use of ancient formulas today [45]. As the saying goes, “the secret of Chinese medicine is in the dosage,” the dosage of Chinese herbs has always been difficult to study. It can be influenced by mistakes in the calculation of the dose in the original historical texts, the production area and effective fraction of Chinese herbs, and the formulation of the preparation. Due to differences of weights and measures between ancient and modern times, it is generally agreed that one “*Liang*” in ancient China is equal to 3 g. Irrespective of *Zhang Zhongjing*'s classical prescriptions or current prescriptions, the dose conversion refers to the criteria used today. This leads to the limitation of doses of many Chinese herbs except for natural minerals of Chinese herbs in the *Pharmacopoeia of the People's Republic of China* (2010 edition). It greatly limits flexible application of drug dose by TCM physicians. Also, some physicians have paid insufficient attention to the dosage problem, especially for monarch drugs such as *Tianma* (Gastrodia), *Chuanxiong* (*Ligusticum Chuanxiong* Hort), *Juhua* (*Chrysanthemum*), *Shigao* (*Gypsum fibrosum*), and *Niuxi* (*Achyranthes* root) in the treatment of hypertension [51]. 

Routine dosages of those monarch drugs in *Pharmacopoeia of the People's Republic of China* (2010 edition) are shown in Table 2. However, in our previous studies, we demonstrated the relationship between Chinese herbs and dosages through the literature review and mining [45, 51]. The result indicated that although routine dosage such as 3 to 10 g could control BP in some extent, higher dosage of certain monarch Chinese herbs may show better antihypertensive effects in hypertension treatment. The dose-effect relationship is obvious in a certain range. It is worth noting that the conclusions have been confirmed in randomized controlled trials of type 2 diabetes in China. And the recommended dosages of those monarch Chinese herbs are also shown in Table 2. 

Taking *Chuanxiong* (*Ligusticum Chuanxiong* Hort) as an example, it is often used in the treatment for hypertension with severe headache. The dose must be greater if the headache is more severe with high BP, whereas the routine dose (3–10 g) is usually ineffective [101]. Also, the recommended dose must be ≥30 g if treating hypertensive crisis and hypertensive encephalopathy [51]. *Niuxi* (*Achyranthes* root) is another important drug often used in the treatment for hypertension with lower-limb swelling and tiredness in the loins and knees. The recommended dose of *Niuxi* (*Achyranthes* root) must be ≥60 g for lowering BP [51], whereas the routine dosage is “5–12 g” [101]. It is also found that the higher the BP, the greater the dosage. *Chuan Niuxi* (*Achyranthes bidentata*) and *Huai Niuxi* (*Achyranthes bidentata* Blume) are used simultaneously when treating hypertensive crisis and hypertensive encephalopathy. Also, the dose of these herbs can start from 30 g to a maximum dose of ≤120 g, respectively [51]. *Tianma* (*Gastrodia*) and *Juhua* (*Chrysanthemum*) are two classical Chinese herbs used for treating hypertension with dizziness. The routine dosages of these two herbs are “3–10 g” and “5–10 g,” respectively [101]. It was also found that the recommended dose of these herbs must be ≥30 g when lowering BP [51, 102]. Therefore, it could be concluded that reasonable dosage of Chinese herbs is closely related to clinical outcomes. However, further randomized, placebo-controlled clinical trials with strict design and adequate sample size are warranted to elaborate the dose-effect relationship and mechanism of Chinese herbs and formulas on treating hypertension. Moreover, due to the certain toxic and adverse effects of these herbs, indications of herbs must be followed strictly [101].

## 6. Discussion and Perspectives

CAM has been frequently used and is gaining popularity worldwide, especially in the most developed countries such as North America, Europe, and Australia. Due to aging of population and prevalence of chronic diseases and stress-related diseases as well as concern about the adverse reaction of chemical drugs, CAM is increasingly popular [103–107]. Recently, Nature has published a special issue of traditional Asian medicine (vol. 480 no. 7378_supp ppS81-S121, 2011). The topic was derived from TCM, also including Kampo medicine and Korean medicine. It traced its origin to today's modernization. As the editorial department comments, it “plays an important role in health maintenance for the peoples of Asia, and is becoming more frequently used in countries in the West” [108]. Some perspectives in the special edition are very insightful and very heuristic. 

The merits of traditional medicine to people's healthcare have been proven by its age-old legacy, its clinical efficacy in curing diseases, and improving quality of life from the ancient times to the present day [109–112]. TCM has long been used in the treatment of a wide variety of illnesses including hypertension. And a series of medical practices were originated including Chinese herbs and formulas, acupuncture, moxibustion, cupping, qigong, Tai Chi (shadow boxing exercise), diet, and exercise therapy [113–116]. Recently, many RCT trials and SRs showed that acupuncture is useful for lowering BP level and improving the circadian rhythm of BP in patients with hypertension [117–120]. Among them, Chinese herbal therapy is the most commonly used. Current researches showed that great progress has been made in Chinese herbs and formulas for the treatment of hypertension from theory, experiments to clinic fields both in vitro and vivo [121–126]. In our previous studies, we also demonstrated the potential effect of Chinese herbs and formulas on decreasing blood pressure, protecting target organs, regulating rennin-angiotensin-aldosterone system, reversing risk factors, and improving endothelial function, although their effects on BP were inferior to CCB, ACEI, ARB, and other common used western medicines [16, 51, 127]. Furthermore, as a special form of Chinese herbs and formulas, Chinese patent drugs are commonly used in the treatment of hypertension in clinic, including Niuhuang Jiangya Pill, QingGan JiangYa Capsule, and Yangxue Qingnao Granule [128, 129]. They have also played an important role in the treatment of hypertension. However, due to the generally low methodological quality and small sample size, single-center of TCM clinical trials, there is little conclusive clinical evidence to support the antihypertensive efficacy of the vast majority of Chinese medicines [8, 130]. Therefore, although the popularity and expenditure of TCM have been increased dramatically, the potential role of TCM in modern hypertension clinical practice and health care system seems to be limited and have even been questioned. 

In the light of our previous studies, it is not difficult to find that the etiology, pathogenesis, and control strategies of hypertension have been changed quietly. The concept highlighting the combination of syndrome and disease, reunderstanding the etiology and pathogenesis, and applying pharmacological achievement, as well as paying attention to dosage, has been gradually and widely accepted, which happens to coincide with the concept and treatment of modern medicine based on biomedical diagnosis. Only by facing these aspects rigorously, the clinical efficacy of Chinese herbs and formulas on hypertension could be improved. What is more, the emergence of evidence-based medicine (EBM) had provided objective therapeutic evaluation of TCM or integrative medicine with new thinking and method [131]. Eugene Braunwald, a famous cardiologist, also pointed out that current cardiology practice is evidence-based and global in scope [132]. Thus, it is urgent to formulate a scientific way to evaluate the efficacy and safety. Unfortunately, due to the poor methodological quality in the majority of current controlled studies, confirmative conclusions on the beneficial effect of TCM for essential hypertension could not be drawn conclusively, and more rigorous trials are needed. We firmly believe that applying these findings to clinical researches will play an important role in hypertension prevention and public health practice. 

Additionally, making good use of these findings and achievements could contribute to modern new drug discovery for hypertension in TCM [16, 133]. Due to the inadequate effectiveness and concerns of safety of current antihypertensive western drugs, a great need has arisen to develop both efficacious and pharmaceutical medicines to fight against this disease. Screening the high efficiency and fewer adverse effects of antihypertensive drugs from Chinese herbs and formulas for hypertension attracts great attention of researchers. From the 1950s, Chinese physicians had been concentrating on effective prevention strategies for hypertension using TCM, and considerable progress had been achieved. Alkaloids of *Rauwolfia verticillata* (the first antihypertensive drug independently invented in China) consist of *reserpine* and ingredients such as *α*-receptor blockers. Total puerarin flavonoids and tetramethylpyrazine are effective components extracted from *Gegen* (*Kudzu* root) and *Chuanxiong* (*Ligusticum chuanxiong* Hort), respectively, by scientists from the Chinese Academy of Medical Sciences; both of which could not only lower BP but also improve signs and symptoms in hypertensive patients [134]. It is noteworthy that due to the unique experience in treating hypertension and its related symptoms in TCM, effective drug screening would be more targeted [135]. The development of modern new drugs for hypertension will be full of challenge and opportunity, but we have full confidence.

## Figures and Tables

**Table 1:** Crucial Chinese herbs in classical formulas recommended for hypertension treatment.

Syndrome	Formulas	Components	TCM efficacy	Crucial Chinese herbs
Fire syndrome	*Tianma Gouteng Yin *(decoction of *Gastrodia *and *Uncaria*)	*Tianma* (*Gastrodia*), *Gouteng* (*Uncaria*), *Shijueming *(*Abalone shell*), *Duzhong *(*Eucommia ulmoides*), *Niuxi* (*Achyranthes *root), *Sangjisheng* (*Loranthus*), *Zhizi* (*Gardenia*), *Huangqin* (*Scutellaria Baicalensis Georgi*), *Yimucao *(*Leonurus japonicus*), *Yejiaoteng* (*Caulis polygoni multiflori*), and *Fushen* (*Poria cocos*).	Suppressing liver yang hyperactivity, clearing heat, activating blood, and nourishing the kidney.	*Tianma* (*Gastrodia*), *Gouteng* (*Uncaria*), *Duzhong *(*Eucommia ulmoides*), and *Niuxi* (*Achyranthes *root).
	*Huanglian Jie Du Tang* (detoxicant decoction of *Coptis*)	*Huanglian* (*Coptis Chinensis*), *Huangqin* (*Scutellaria Baicalensis Georgi*), *Huangbai *(*Phellodendron *bark), and *Zhizi* (*Gardenia*).	Counteracting heart fire syndrome, clearing heat and toxic materials, and relieving headache and dizziness.	*Huanglian* (*Coptis Chinensis*), *Huangqin* (*Scutellaria Baicalensis Georgi*), *Huangbai *(*Phellodendron *bark), and *Zhizi* (*Gardenia*).
	*Zeng Ye Tang *(fluid-increasing decoction)	*Xuanshen* (*Radix scrophulariae*), *Maidong* (*Dwarf lilyturf tuber*), and *Shengdihuang* (*Radix rehmanniae*).	Counteracting stomach fire and intestinal fire syndrome and nourishing yin to relieve the symptoms of dryness.	*Xuanshen* (*Radix scrophulariae*) and *Shengdihuang* (*Radix rehmanniae*).

Fluid retention syndrome	*Wuling* powder	*Fuling *(*Poria cocos*),* Guizhi *(*Cassia twig*), *Zhuling* (*Polyporus*), *Zexie *(*Alisma*), and *Baizhu* (*Atractylodes*).	Removing dampness by promoting dieresis.	*Zexie *(*Alisma*) and *Baizhu* (*Atractylodes*).
	*Zexie Tang *(decoction of *Alisma*)	*Zexie *(*Alisma*) and *Baizhu* (*Atractylodes*).	Counteracting fluid retention syndrome.	*Zexie *(*Alisma*) and *Baizhu* (*Atractylodes*).
	*Banxia Baizhu Tianma Tang *(decoction of *Pinellia ternata*, *Atractylodes macrocephala*, and *Gastrodia elata*)	*Banxia *(*Pinellia ternate*), *Baizhu* (*Atractylodes*), *Tianma* (*Gastrodia*), *Chenpi *(*Tangerine peel*), *Fuling *(*Poria cocos*), *Gancao *(*Glycyrrhiza*), *Shengjiang* (*Ginger*), and *Hongzao* (*Red jujube*).	Calming the liver, strengthening the spleen, and dissipating excessive fluid.	*Tianma* (*Gastrodia*).

Deficiency syndrome	*Liu Wei Dihuang Wan *(pill of *Rehmannia*)	*Dihuang* (*Radix rehmanniae*), *Shanyurou *(*Pulp of cornus*), *Shanyao* (*Yam*), *Fuling *(*Poria cocos*), *Zexie *(*Alisma*), and *Danpi *(*Cortex moutan*).	Replenishing kidney yin.	*Dihuang* (*Radix rehmanniae*) and *Danpi *(*Cortex moutan*).
	*Shen qi Wan* (kidney *qi* pill)	*Guizhi *(*Cassia twig*), *Fuzi* (*Prepared aconite root*), *Dihuang* (*Radix rehmanniae*), *Shanyurou *(*Pulp of cornus*), *Shanyao* (*Yam*), *Fuling *(*Poria cocos*), *Zexie *(*Alisma*), and *Danpi *(*Cortex moutan*).	Recuperating the kidney yang.	*Dihuang* (*Radix rehmanniae*) and *Danpi *(*Cortex moutan*).

**Table 2:** Drug dosage of crucial Chinese herbs recommended for hypertension treatment.

Chinese herbs	TCM efficacy	Indications	Routine dosage	Recommended dosage
*Chuanxiong* (*Ligusticum Chuanxiong* Hort)	Promoting *qi*, activating blood circulation, and relieving pain.	Hypertension with severe headache, hypertensive crisis, and hypertensive encephalopathy.	3–10 g	≥30 g

*Niuxi* (*Achyranthes* root)	Nourishing liver and kidney, strengthening muscles and bones, promoting blood circulation to remove blood stasis, making fire and blood downstream, promoting diuresis, and relieving stranguria.	Hypertension with lower-limb swelling and tiredness in the loins and knees.	5–12 g	60–120 g

*Tianma* (*Gastrodia*)	Calming liver, suppressing liver-yang hyperactivity, activating collaterals, and relieving spasm.	Hypertension with dizziness.	3–10 g	≥30 g

*Juhua* (*Chrysanthemum*)	Clearing away heat, dispelling wind, calming the liver, and improving eyesight.	Hypertension with dizziness.	5–10 g	≥30 g
